# Nontuberculous mycobacterial pulmonary disease added burden to COPD and bronchiectasis in Japan

**DOI:** 10.1183/23120541.00911-2023

**Published:** 2024-07-08

**Authors:** Ping Wang, Kozo Morimoto, Naoki Hasegawa, Mariam Hassan, Anjan Chatterjee

**Affiliations:** 1 Insmed Incorporated, Bridgewater, NJ, USA; 2 Fukujuji Hospital, Japan Anti-Tuberculosis Association, Tokyo, Japan; 3 Keio University Hospital, Tokyo, Japan

## Abstract

**Background and objective:**

Nontuberculous mycobacterial pulmonary disease (NTM-PD) prevalence in Japan is among the highest worldwide. COPD and bronchiectasis are common comorbidities among patients with NTM-PD, and it is challenging to treat NTM-PD in patients with these conditions. There are limited data on the incremental burden that NTM-PD adds to underlying COPD or bronchiectasis in Japan. Therefore, the objective of this study was to assess the incremental burden associated with NTM-PD in patients with pre-existing COPD and/or bronchiectasis.

**Methods:**

This nested case–control study was based on JMDC, Inc. claims data (2015–2020). Patients with COPD and/or bronchiectasis with NTM-PD (cases) were age and sex matched 1:3 to patients with COPD and/or bronchiectasis without NTM-PD (controls), resulting in three mutually exclusive patient groups (COPD, bronchiectasis or both; with or without NTM-PD). Incremental burden of NTM-PD was assessed within each group by comparing hospitalisations during the 1-year period after NTM-PD diagnosis (index) between cases and controls with both univariate analysis and multivariate analysis adjusting for pre-index comorbidities.

**Results:**

Univariate analyses in the three patient groups consistently demonstrated incremental hospitalisation burden in cases *versus* controls (*e.g.* COPD group: 20% of 492 cases *versus* 13% of 1476 controls had all-cause hospitalisations; 11% *versus* 5% had respiratory-related hospitalisations; and 6% *versus* 2% had COPD-related hospitalisations). Subsequent multivariate analysis further confirmed the findings.

**Conclusions:**

The substantial incremental burden of hospitalisation associated with NTM-PD in patients with COPD and/or bronchiectasis highlights the urgent need for appropriate management of NTM-PD in Japan.

## Shareable abstract

**NTM-PD is a progressive, debilitating lung infection. This nested case–control study found NTM-PD added substantial hospitalisation burden to pre-existing COPD and/or bronchiectasis in Japan, highlighting the urgent need for appropriate NTM-PD management.** https://bit.ly/49Gr7YH

## Introduction

Nontuberculous mycobacterial pulmonary disease (NTM-PD) is a progressive and debilitating pulmonary infection associated with increased healthcare utilisation [1–3]. Prevalence of NTM-PD varies globally [4] with reported increases in the USA, Germany and Korea [5–7]. The estimated prevalence rate of NTM-PD in Japan is among the highest reported: 29 per 100 000 persons (2011) [8]. The estimated incidence of NTM-PD in Japan has increased from ∼5.7 per 100 000 person-years in 2007 to 14.7 per 100 000 person-years in 2014) [9]. The markedly higher prevalence relative to incidence estimates may be due to NTM-PD being chronic and difficult to treat [2, 8].

Patients with NTM-PD often have comorbid respiratory conditions such as COPD and bronchiectasis [1, 10–12]. In an administrative claims-based analysis, patients with NTM-PD were six times more likely to have comorbid COPD than those without [13]. Prevalence of bronchiectasis in Japan is unclear [14]; however, the frequency of comorbid bronchiectasis among patients with NTM-PD ranges from <5% to >80% [2, 8, 10, 15]. Such large variations may be attributable to low reports of bronchiectasis by physicians in insurance claims but much higher rates of bronchiectasis confirmed by chest images available in hospital records [2, 8, 10, 15].

Diagnosis of NTM-PD may be delayed [16–18], particularly in patients with pre-existing lung diseases, such as COPD or bronchiectasis, due to nonspecific, overlapping symptoms (*e.g.* cough or shortness of breath) [1, 16–20]. Delays in diagnosis and treatment of NTM-PD in patients with coexisting lung diseases can contribute to disease progression and poor clinical outcomes [21, 22]. Notably, current international clinical practice guidelines for NTM-PD recommend timely treatment initiation rather than “watchful waiting”, especially for patients with positive acid-fast bacilli sputum smears and/or cavitary lung disease [23].

US-based investigations have reported that NTM-PD has been associated with significantly higher hospitalisation burden among patients with underlying COPD or bronchiectasis [3, 24]; however, the incremental burden that NTM-PD may add to pre-existing COPD and/or bronchiectasis in Japan is under-researched. The objective of this study was to assess the incremental burden that NTM-PD adds to patients with pre-existing COPD, bronchiectasis, or concomitant bronchiectasis and COPD in Japan by comparing hospitalisations between patients with NTM-PD *versus* those without NTM-PD. Such fine segmentation of the patient population may allow more precise characterisation of the burden of NTM-PD in these high-risk patients and enable appropriate healthcare resource allocation and timely management of NTM-PD.

## Methods

### Data source

This nested case–control study was conducted using claims data provided by the JMDC, Inc. (formerly Japan Medical Data Centre) from 1 February 2015 to 29 February 2020 (representing 6 504 929 lives in total). The JMDC contracts with multiple Japanese health insurance societies and has accumulated reimbursement data, consisting of receipts (inpatient, outpatient and dispensing) and medical examination data, from enrolled employees and their dependents (age range, from birth to 74 years) [25]. Ethics approval was not required based on the Ethical Guidelines for Medical and Biological Research Involving Human Subjects [26] since only publicly available de-identified and encrypted data were used [25].

### Study population

JMDC database members with NTM-PD and pre-existing comorbid COPD and/or bronchiectasis were identified as meeting the following requirements: 1) a diagnosis of NTM-PD; and 2) a diagnosis of either COPD, both COPD and bronchiectasis, or bronchiectasis alone, before the first diagnosis of NTM-PD. All eligible patients with COPD and/or bronchiectasis and NTM-PD (cases) were matched 1:3 to patients with COPD and/or bronchiectasis without NTM-PD (controls) by age on index date and sex, forming three mutually exclusive case–control patient groups (table 1).

**TABLE 1:** Definitions of case patients and control patients in the three mutually exclusive case–control patient groups for assessing incremental burden of nontuberculous mycobacterial pulmonary disease (NTM-PD)

	Patient groups		
	COPD	Bronchiectasis and COPD	Bronchiectasis
**Cases^#^: NTM-PD**	Pre-existing COPD^¶^ No bronchiectasis	Pre-existing bronchiectasis^¶^ Pre-existing COPD^¶^	Pre-existing bronchiectasis^¶^ No COPD
**Controls^#^: no NTM-PD**	COPD No bronchiectasis	Bronchiectasis and COPD	Bronchiectasis No COPD

^#^: within each mutually exclusive case–control patient group, cases were matched 1:3 to age- and sex-matched controls; ^¶^: COPD and/or bronchiectasis preceded index date (*i.e.* date that the first claim with NTM-PD diagnosis (International Classification of Diseases, 10th revision, A31.0 and A31.9) was received).

The index date for cases was defined as date of first medical claim with a diagnosis of NTM-PD. The index date for controls was a uniformly distributed random date after diagnosis of COPD and/or bronchiectasis. All cases and matched controls had continuous health insurance coverage from 1 year pre-index to 1 year post-index.

### Definitions of patients with NTM-PD, COPD and bronchiectasis

A patient with NTM-PD was defined as an individual with two or more claims with a diagnostic code for NTM-PD (International Classification of Diseases, 10th Revision, Clinical Modification (ICD-10-CM) A31.0 and A31.9) in two different months but within 12 months, from ambulatory or inpatient encounters [5, 27]. A patient with COPD was defined as an individual who had either two or more ambulatory encounters with a diagnostic code for COPD (ICD-10-CM J41–J44) in two different months, or one or more hospitalisations with a diagnostic code for COPD [28]. A patient with bronchiectasis was defined as an individual with either two or more ambulatory encounters with a diagnostic code for bronchiectasis (ICD-10-CM J47) in two different months, or one or more ambulatory encounter with a bronchiectasis diagnosis and a computed tomography scan of the thorax (Current Procedural Terminology 4 codes 71250, 71260 and 71270) within 2 months before the encounter, or one or more inpatient claims with a diagnostic code for bronchiectasis [28]. The identification of patients with NTM-PD, COPD and bronchiectasis was similar to validated approaches based on ICD-10-CM or ICD-9-CM codes [5, 27, 28].

### Study outcomes

Over the 1-year post-index follow-up period, all-cause, respiratory-related, COPD-related and bronchiectasis-related hospitalisations were compared between cases and controls by: 1) proportions of patients who were hospitalised; and 2) annual hospitalisation rate (number of hospitalisations per patient per year (PPPY)). Respiratory-related hospitalisations were defined as hospitalisations with a diagnosis for respiratory conditions (ICD-10-CM J00–J99). Likewise, COPD- and bronchiectasis-related hospitalisations were defined as those with diagnosis for COPD and bronchiectasis, respectively.

### Statistical analysis

Descriptive analyses of demographics including age at index date, sex and insured status as well as clinical characteristics during the 1-year pre-index period were conducted for patients with both COPD and/or bronchiectasis and NTM-PD (cases) and matched patients with COPD and/or bronchiectasis without NTM-PD (controls).

The incremental burden of hospitalisation associated with NTM-PD in patients with pre-existing COPD and/or bronchiectasis was analysed with univariate analysis of hospitalisations accrued over the 1-year post-index follow-up period, including 1) proportion of patients hospitalised and 2) number of hospitalisations PPPY, followed with multivariate analyses controlling for confounding comorbidities during the 1-year pre-index period, including logistic regression for estimating the adjusted odds ratio of hospitalisation and Poisson regression for estimating the adjusted hospitalisation incidence rate ratio (IRR). Comorbidities were identified based on a single occurrence of corresponding diagnostic ICD-10-CM codes in any medical claim (table S1).

Categorical variables are presented as the number and proportion of patients; continuous variables are summarised by mean and standard deviation, median, and quartiles. Statistical tests comparing the case *versus* control groups included McNemar's test for categorical variables and the Wilcoxon signed-rank test for continuous variables. An α=0.05 was considered statistically significant.

## Results

### Study population

We identified 492 case patients with COPD with NTM-PD, 37 with bronchiectasis and COPD with NTM-PD, and 53 with bronchiectasis with NTM-PD. Each of these case patients was matched by age and sex to 1476 control patients with COPD without NTM-PD, 111 with bronchiectasis and COPD without NTM-PD, and 159 with bronchiectasis without NTM-PD (figure S1).

### Demographics and pre-index clinical characteristics

Age at index date (mean±sd 56.6±10.3, 58.1±9.5 and 58.5±9.4 years in the COPD, bronchiectasis and COPD, and bronchiectasis groups, respectively) (table 2) and time between diagnosis of COPD/bronchiectasis and subsequent diagnosis of NTM-PD (approximately 1 year) were similar in all groups.

**TABLE 2:** Patient demographics and clinical characteristics during the 1-year pre-index period

	COPD group		Bronchiectasis and COPD group		Bronchiectasis group	
	Case: with NTM-PD	Control: without NTM-PD	Case: with NTM-PD	Control: without NTM-PD	Case: with NTM-PD	Control: without NTM-PD
**Subjects**	492	1476	37	111	53	159
**Age at index date, years, mean±sd**	56.6±10.3	56.6±10.3	58.1±9.5	58.1±9.4	58.5±9.4	58.5±9.4
**Female**	302 (61.4)	906 (61.4)	28 (75.7)	84 (75.7)	42 (79.2)	126 (79.2)
**Insured status**						
Insured employee	265 (53.9)	837 (56.7)	14 (37.8)	48 (43.2)	22 (41.5)	70 (44.0)
Employee dependent	227 (46.1)	639 (43.3)	23 (62.2)	63 (56.8)	31 (58.5)	89 (56.0)
**Selected nonpulmonary comorbidities**						
All cardiovascular diseases^#^	210 (42.7)	834 (56.5)	11 (29.7)	51 (46.0)	20 (37.7)	67 (42.1)
GORD^#^	173 (35.2)	541 (36.7)	10 (27.0)	35 (31.5)	15 (28.3)	38 (23.9)
Hypertension^#^	120 (24.4)	649 (44.0)	6 (16.2)	39 (35.1)	13 (24.5)	46 (28.9)
All cancers, excluding lung cancer^#^	87 (17.7)	193 (13.1)	2 (5.4)	12 (10.8)	7 (13.2)	15 (9.4)
Diabetes mellitus^#^	40 (8.1)	146 (9.9)	2 (5.4)	7 (6.3)	5 (9.4)	15 (9.4)
Chronic kidney disease^#^	5 (1.0)	42 (2.8)	0 (0.0)	3 (2.7)	0 (0)	3 (1.9)
Dementia^#^	4 (0.8)	22 (1.5)	0 (0.0)	2 (1.8)	0 (0.0)	0 (0.0)
Overweight and obesity^#^	2 (0.4)	31 (2.1)	0 (0.0)	1 (0.9)	1 (1.9)	0 (0)
**Selected pulmonary symptoms**						
Haemoptysis	54 (11.0)	25 (1.7)	7 (18.9)	10 (9.0)	10 (18.9)	13 (8.2)
Cough	27 (5.5)	57 (3.9)	5 (13.5)	6 (5.4)	1 (1.9)	6 (3.8)
Dyspnoea	8 (1.6)	9 (0.6)	2 (5.4)	1 (0.9)	0 (0)	1 (0.63)
**Selected pulmonary comorbidities**						
Asthma^#^	182 (37.0)	652 (44.2)	10 (27.0)	48 (43.2)	17 (32.1)	55 (34.6)
Pneumonia	154 (31.3)	245 (16.6)	14 (37.8)	28 (25.2)	20 (37.7)	67 (24.5)
Emphysema	64 (13.0)	187 (12.7)	3 (8.1)	6 (5.4)	0 (0.0)	0 (0.0)
Malignant neoplasm of bronchus and lung	34 (6.9)	55 (3.7)	0 (0.0)	3 (2.7)	1 (1.9)	1 (0.6)
Idiopathic interstitial lung disease^#^	26 (5.3)	54 (3.7)	4 (10.8)	9 (8.1)	0 (0)	4 (2.5)
**Patients with annual health check-up record**	311	927	24	62	30	105
Habitual smoker based on annual health check-up^¶^	41 (13.2)	207 (22.3)	0 (0.0)	11 (18.0)	1 (3.3)	7 (6.7)

Data are presented as n or n (%), unless otherwise stated. Comorbidities that were not significantly different between cases and controls (p≥0.05): pneumonia, gastro-oesophageal reflux disease (GORD), and hypertension in the bronchiectasis and COPD group and the bronchiectasis group, and asthma in the bronchiectasis group; all other comorbidities with non-zero patient counts were significantly different between cases and controls in each group; p-values met the statistical significance level (α=0.05) based on McNemar's test. NTM-PD: nontuberculous mycobacterial pulmonary disease. ^#^: comorbidities included in the multivariate analyses. ^¶^: habitual smokers were identified using annual health check-up records during the 1-year pre-index period. Not all patients had annual check-up records. The percentages for habitual smokers were calculated based on the number of patients with annual check-up records. Statistical difference was tested with the Chi-squared test to accommodate the fact that only a subset of the matched patients had annual check-up data and a statistically significant difference was observed between cases and controls in the COPD group (p<0.05).

Clinical characteristics were evaluated during the 1-year pre-index period (table 2). Comorbidities that were consistently less common in cases with NTM-PD *versus* controls without NTM-PD included all cardiovascular diseases (42.7% *versus* 56.5% in the COPD group; 29.7% *versus* 46.0% in the bronchiectasis and COPD group; 37.7% *versus* 42.1% in the bronchiectasis group; all p<0.05), hypertension (24.4% *versus* 44.0% in the COPD group, p<0.05; 16.2% *versus* 35.1% in the bronchiectasis and COPD group, p>0.05; 24.5% *versus* 28.9% in the bronchiectasis group, p>0.05) and diabetes (8.1% *versus* 9.9% in the COPD group, 5.4% *versus* 6.3% in the bronchiectasis and COPD group, 9.4% *versus* 9.4% in the bronchiectasis group; all p<0.05). Gastro-oesophageal reflux disease (GORD) had no statistically significant difference between cases and controls in the bronchiectasis and COPD (27.0% *versus* 31.5%) and bronchiectasis (28.3% *versus* 23.9%) groups. GORD was more common among patients with COPD (cases 35.2% *versus* controls 36.7%; p<0.05) *versus* those with bronchiectasis, regardless of the presence of NTM-PD.

Pulmonary symptoms and comorbidities generally occurred in a higher proportion of cases compared with controls in each patient group except asthma (table 2). Asthma was the most common pulmonary comorbidity overall and was consistently lower among cases than controls in all patient groups (37.0% *versus* 44.2% in the COPD group, p<0.05; 27.0% *versus* 43.2% in the bronchiectasis and COPD group, p<0.05; 32.1% *versus* 34.6% in the bronchiectasis group, p>0.05). Haemoptysis was more common in cases than controls across all groups and was more prevalent among patients with comorbid bronchiectasis (11.0% *versus* 1.7% in the COPD group, p<0.05; 18.9% *versus* 9.0% in the bronchiectasis and COPD group, p<0.05; 18.9% *versus* 8.2% in the bronchiectasis group, p<0.05). Pneumonia was more common in cases than in controls across groups and more prevalent among patients with comorbid bronchiectasis (31.3% *versus* 16.6% in the COPD group, p<0.05; 37.8% *versus* 25.2% in the bronchiectasis and COPD group, p>0.05; 37.7% *versus* 24.5% in the bronchiectasis group, p>0.05).

### Incremental hospitalisation burden associated with NTM-PD in patients with COPD

#### Proportion of patients with hospitalisations

**FIGURE 1:**
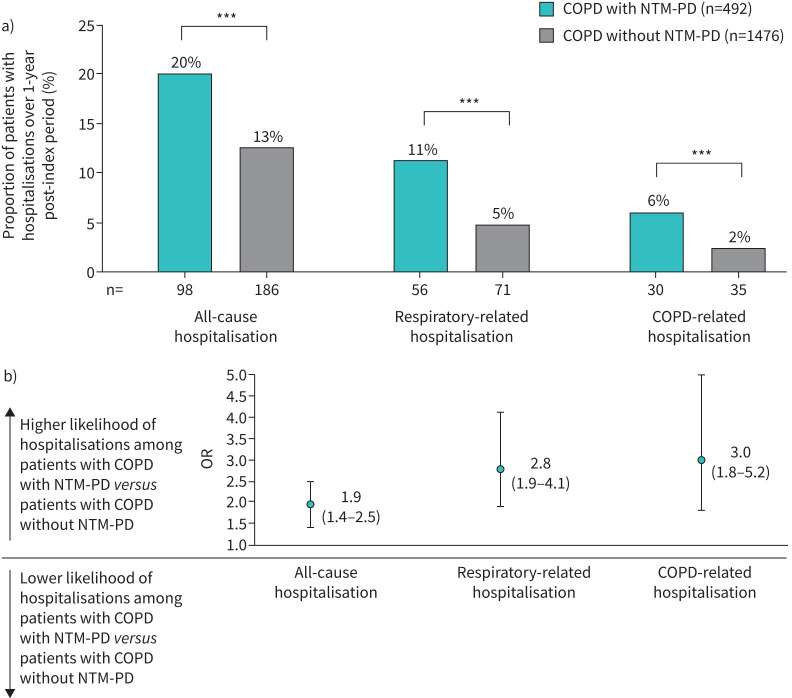
a) Proportion of patients with hospitalisations over a 1-year follow-up period in patients with COPD and NTM-PD *versus* matched patients with COPD without NTM-PD and b) odds ratios associated with nontuberculous mycobacterial pulmonary disease (NTM-PD) after multivariate analysis adjustment (ORs derived from logistic regression analysis controlling for comorbidities during the 1-year pre-index period: diabetes mellitus; hypertension; overweight and obesity; chronic kidney disease; total cardiovascular diseases; cancer, excluding lung; dementia; gastro-oesophageal reflux disease; asthma; idiopathic pulmonary fibrosis; and idiopathic interstitial lung disease). Data are presented as a) n (%) and b) OR (95% CI). ***: p<0.001 based on McNemar's test for differences between cases and controls.

Univariate analysis found that a significantly higher proportion of patients with COPD with NTM-PD had hospitalisations than matched controls with COPD without NTM-PD: 20% of cases *versus* 13% of controls experienced an all-cause hospitalisation, 11% *versus* 5% a respiratory-related hospitalisation, and 6% *versus* 2% a COPD-related hospitalisation (all p<0.001) (figure 1a).

After multivariate logistic regression analysis controlling for pre-index confounding comorbidities, patients with COPD with NTM-PD were OR 1.9 (95% CI 1.4–2.5), 2.8 (95% CI 1.9–4.1) and 3.0 (95% CI 1.8–5.2) times more likely to experience an all-cause, respiratory-related, and COPD-related hospitalisation, respectively (figure 1b).

#### Annualised hospitalisation rates

**FIGURE 2:**
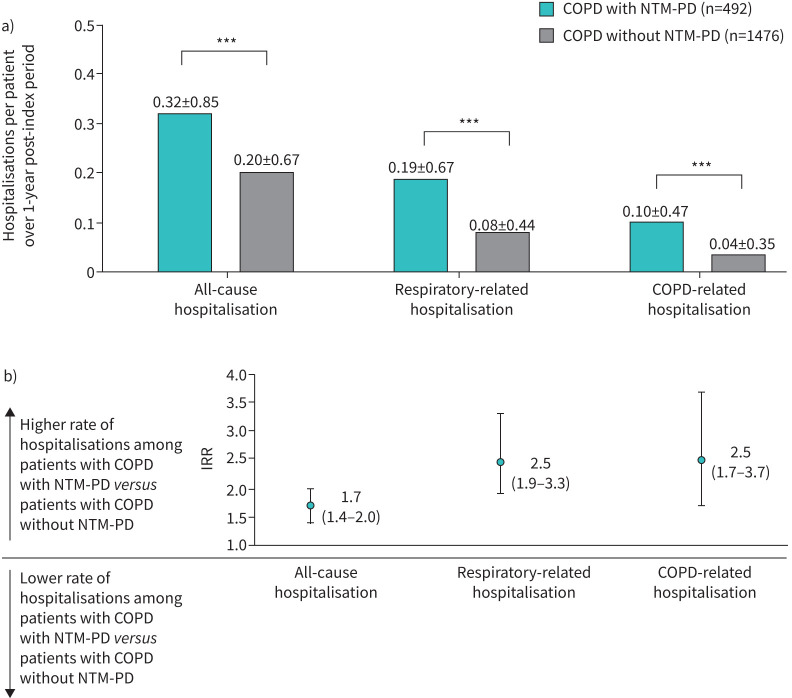
a) Per-patient rates of hospitalisation over a 1-year follow-up period in patients with COPD and NTM-PD *versus* matched patients with COPD without NTM-PD and b) incidence rate ratios (IRRs) associated with nontuberculous mycobacterial pulmonary disease (NTM-PD) after multivariate analysis adjustment (IRR is a ratio of incidence rate between the two groups being compared; IRR values derived from Poisson regression analysis controlling for comorbidities during the 1-year pre-index period: diabetes mellitus; hypertension; overweight and obesity; chronic kidney disease; total cardiovascular diseases; cancer, excluding lung; dementia; gastro-oesophageal reflux disease; asthma; idiopathic pulmonary fibrosis; and idiopathic interstitial lung disease). Data are presented as a) mean±sd and b) IRR (95% CI). ***: p<0.001 based on Wilcoxon signed-rank test for differences between cases and controls.

Univariate analysis found a significantly higher hospitalisation rate among patients with COPD with NTM-PD than among controls with COPD without NTM-PD (figure 2a). Mean±sd hospitalisations per patient over the 1-year post-index period in cases *versus* controls was 0.19±0.67 *versus* 0.08±0.44 (p<0.001) for respiratory-related hospitalisations. Similar statistically significant differences were observed for all-cause and COPD-related hospitalisations (p<0.001) (figure 2a).

After multivariate Poisson regression analysis controlling for confounding pre-index comorbidities, patients with COPD and NTM-PD had IRR 1.7 (95% CI 1.4–2.0), 2.5 (95% CI 1.9–3.3) and 2.5 (95% CI 1.7–3.7) times the rate of all-cause, respiratory-related and COPD-related hospitalisations (figure 2b).

A subgroup analysis among hospitalised patients in the COPD group during the 1-year post-index follow-up further examined the impact of NTM-PD in hospitalised patients with COPD. There were no significant differences between cases and controls in either number of admissions per patient (mean 1.6, p=0.6) or length of stay per patient (mean 12–13 days, p=0.4) (table S2).

### Incremental hospitalisation burden associated with NTM-PD in patients with bronchiectasis

#### Proportion of patients with hospitalisations

**FIGURE 3:**
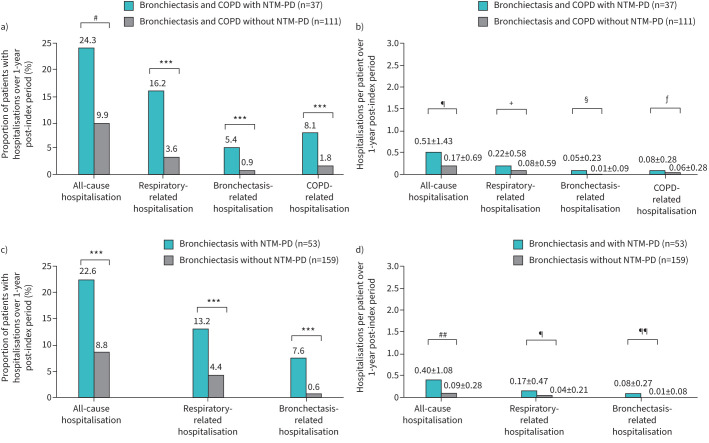
a) Proportion of patients with hospitalisations* and b) per-patient rates of hospitalisation^‡^ in patients with bronchiectasis and COPD with nontuberculous mycobacteriual pulmonary disease (NTM-PD) *versus* matched patients with bronchiectasis and COPD without NTM-PD; and c) proportion of patients with hospitalisations* and d) per-patient rates of hospitalisation^‡^ in patients with bronchiectasis with NTM-PD *versus* matched patients with bronchiectasis without NTM-PD in Japan over a 1-year follow-up period. Data are presented as a and c) percentages or b and d) mean±sd. p-values represent between-group comparisons based on a and c) McNemar's test and b and d) Wilcoxon signed-rank test. ***: p<0.001; ^#^: p<0.02; ^¶^: p<0.03; ^+^: p<0.009; ^§^: p=0.095; ^ƒ^: p=0.071; ^##^: p<0.006; ^¶¶^: p<0.005.

Univariate analysis found that a significantly higher proportion of patients with bronchiectasis and COPD with NTM-PD experienced hospitalisations than matched controls with bronchiectasis and COPD without NTM-PD: 24.3% *versus* 9.9% for all-cause, 16.2% *versus* 3.6% for respiratory-related, 5.4% *versus* 0.9% for bronchiectasis-related, and 8.1% *versus* 1.8% for COPD-related hospitalisations (all p<0.05) (figure 3a). Similarly, among patients with bronchiectasis with NTM-PD, the proportion with hospitalisations was significantly greater than that of matched controls with bronchiectasis without NTM-PD for all hospitalisation categories evaluated (all p<0.001) (figure 3c).

#### Annualised hospitalisation rates

Univariate analysis found that all-cause and respiratory-related hospitalisation rates were significantly higher in patients with bronchiectasis and COPD with NTM-PD than in those with bronchiectasis and COPD without NTM-PD (both p<0.05) (figure 3b).

Among patients with bronchiectasis with NTM-PD, the hospitalisation rates were significantly higher for all-cause, respiratory-related, and bronchiectasis-related hospitalisations compared with matched controls with bronchiectasis without NTM-PD (all p<0.05) (figure 3d).

## Discussion

This study shows that patients in Japan with NTM-PD and pre-existing COPD and/or bronchiectasis were significantly more likely to experience hospitalisation and had higher rates of hospitalisation than patients with COPD and/or bronchiectasis without NTM-PD.

Our findings are comparable with a retrospective US Medicare claims database study (2010–2017) that evaluated incremental healthcare resource utilisation in 4010 patients with COPD with NTM-PD and 12 030 age/sex-matched controls with COPD without NTM-PD [3]. That study found patients with COPD and NTM-PD were approximately two times more likely to experience an all-cause, respiratory-related and COPD-related hospitalisation than those with COPD without NTM-PD [3].

A German population-based cohort study found hospitalisation burden among patients with NTM-PD was three times higher than in age/sex/Charlson Comorbidity Index-matched controls without NTM-PD; mortality risk was four-fold higher over the 39-month follow-up period [29]. The mortality rate in patients with COPD with NTM-PD (41.5%; 27/65) was >2 times higher than in patients with COPD without NTM-PD (15.9%; 62/390) [29]. The difference from our study was that it did not require patients to have COPD before their diagnosis of NTM-PD. In addition, a Japan-based analysis of 204 patients with NTM-PD in Nagasaki found that almost half (44.4%; 20 out of 45) of patients with NTM-PD who experienced clinical deterioration within 1 year had underlying lung disease, including COPD [10]. Collectively, these data underscore the need for timely and appropriate management of NTM-PD in high-risk patients with underlying lung disease.

The observed comparable burden of hospitalisation in the subgroup analysis between hospitalised cases and controls in the COPD group may reflect that patients who experienced hospitalisations might have had similar COPD severity; it is also possible that, once hospitalised, similar patterns of patient management were used in Japan, regardless of NTM-PD status.

During the 1-year period prior to the diagnosis of NTM-PD, the study found a consistently lower prevalence of asthma among the cases with NTM-PD compared with the controls without NTM-PD, in all the three patient groups; the difference was statistically significant in the COPD group and the COPD and bronchiectasis group. Similar results were reported from an analysis of patients with bronchiectasis enrolled in the US Bronchiectasis Research Registry, in which asthma was less common in patients with NTM-PD than without NTM-PD (25.7% *versus* 32.5%) [30]. In contrast, a US study that analysed claims data from patients aged ≥65 years reported a higher prevalence of asthma in patients with COPD with NTM-PD than in patients with COPD without NTM-PD (26.5% *versus* 17.3%) [31]. It has been reported that approximately 15–55% of patients may have asthma–COPD overlap syndrome, with variation seen by sex and age [32]. The prevalences of asthma in the different patient groups in our study were within the above range. The varying prevalence of comorbid asthma reported in the literature, as well as in our study, may be a combined effect of the differences in ages, race, and underlying respiratory diseases and their severities, as well as possible misdiagnosis of those conditions. Further investigations are needed to better understand the relationship between asthma, COPD, bronchiectasis and NTM-PD in Japan.

In agreement with previous studies, GORD was a common comorbidity observed in patients with COPD and/or bronchiectasis with or without NTM-PD in this analysis [13, 33–35]. The observation that GORD was more common in patients with COPD than with bronchiectasis, regardless of the presence of NTM-PD, may be related to the higher proportion of male patients and smokers in the COPD group, as both male sex and a history of smoking have been shown to be risk factors for GORD [13, 36].

Claims-based epidemiological research has inherent limitations, including lack of potentially pertinent clinical information in the claims database for confirming NTM-PD [12, 37, 38]. Variations in coding practices in Japan's healthcare systems might also affect patients’ clinical characteristics, such as the lower prevalence of cough and dyspnoea in this study than those in previous studies (ranging from 55% to 96%) [18, 39, 40]. Nevertheless, such differences in documenting the pulmonary symptoms in claims likely apply to both cases and controls and so should not affect our findings regarding the incremental burden associated with NTM-PD in Japan. Prevalence of COPD in Japan has been estimated to be approximately 10–15% in the general population and >30% among high-risk patients [41, 42], but underdiagnosis is well documented [41, 43, 44]. In this study, COPD was identified in <5% of the JMDC population, but any potential underdiagnosis likely affected both the case and control groups comparably. Some of the control group could be complicated with undiagnosed NTM-PD but this cannot be confirmed.

Hospitalisation for COPD exacerbation is positively correlated to COPD severity [45, 46]; it is possible patients with NTM-PD had more severe underlying COPD. However, COPD severity was not assessed due to key indicators [47] not being available in the claims data. Patient numbers in the bronchiectasis groups were small, precluding multivariable adjustment. Nonetheless, NTM-PD was associated with an incremental burden of hospitalisation in both bronchiectasis groups. Findings from the JMDC database are specific to individuals aged <75 years and might not apply to the older population in Japan, nor be generalisable to other countries [38].

This analysis provides the first large-scale population-based estimate of incremental hospitalisation burden associated with NTM-PD in patients with COPD and/or bronchiectasis in Japan. Since COPD and bronchiectasis are risk factors and common comorbidities of patients with NTM-PD, segmentation of patients with NTM-PD into mutually exclusive groups with either COPD, concomitant bronchiectasis and COPD, or bronchiectasis offers insights for better disease management. These findings add to prior studies that have highlighted burdens associated with NTM-PD, such as economic losses owing to the inability to work full-time and costs of long-term medication, as well as an increased risk of mortality [29, 31, 48]. The evidence from this study should help healthcare policy decision makers in Japan who need information on disease burden when making decisions on new medicines. Possible strategies that could enhance the current disease management include increasing the awareness of NTM-PD among physicians and patients in this subpopulation; encouraging early screening and diagnosis with sputum culture and chest imaging; initiating treatment rather than a watchful waiting approach; and maintaining patients on antibiotic treatment for ≥1 year after sputum culture conversion based on the guideline recommendations [23].

In conclusion, the substantial incremental hospitalisation burden associated with NTM-PD in patients with pre-existing COPD and/or bronchiectasis highlights the urgent need for appropriate management of NTM-PD in this subpopulation.

## Supplementary material

10.1183/23120541.00911-2023.Supp1 **Please note:** supplementary material is not edited by the Editorial Office, and is uploaded as it has been supplied by the author.
Supplementary material 00911-2023.SUPPLEMENT

## Data Availability

statement: The data that support the findings of this study are included within the article (and its supplementary material).
