# Supplementary material for: Nontuberculous mycobacterial pulmonary disease added burden to COPD and bronchiectasis in Japan
Source: ERJ Open Res. 2024 Jul 8;10(4):00911-2023. doi: 10.1183/23120541.00911-2023 (PMC11228608; doi:10.1183/23120541.00911-2023)
Supplement: Supplementary file 1 [file 00911-2023.SUPPLEMENT.pdf]

Supplementary materials

Figure S1 Study population identification from the JMDC database: COPD patient group (A) Bronchiectasis and COPD patient group (B) and Bronchiectasis patient group (C)

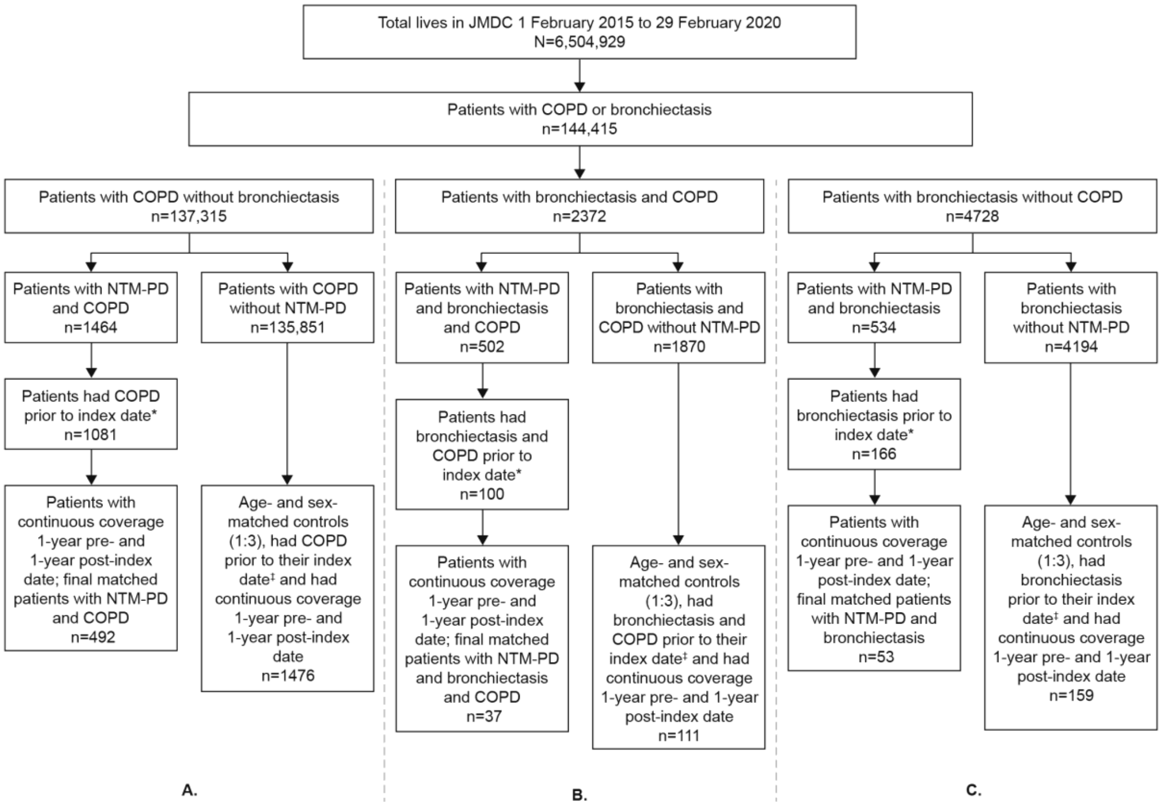

*Index date: the date that the first claim with NTM-PD diagnosis (*ICD-10* A31.0, A31.9) was received for the patients with NTM-PD.

‡Index date: a uniformly distributed random date after diagnosis of COPD and/or bronchiectasis for the patients without NTM-PD.

COPD, chronic obstructive pulmonary disease; *ICD-10-CM*, International Classification of Diseases, 10th Revision, Clinical Modification; JMDC, JMDC, Inc. (formerly Japan Medical Data Center); NTM-PD, nontuberculous mycobacterial pulmonary disease.

Table S1 *ICD-10-CM* diagnostic codes for comorbidities and symptoms

**Comorbidities and symptoms***	***ICD-10-CM***
Non-pulmonary	
All cancers, excluding lung cancer	C00-C33, C37-C96
All cardiovascular diseases	I00-I99
Chronic kidney disease	N18
Dementia	F00, F01, F02, F03, F05, G30, G31, G20
Diabetes mellitus	E10, E11
Gastroesophageal reflux disease	K21
Hypertension	I10-I15
Overweight and obesity	E66
Pulmonary	
Asthma	J45
Cough	R05
Dyspnoea	R060
Emphysema	J43
Haemoptysis	R04
Idiopathic interstitial lung disease	J8410, J8489, J8401, J8403, J8402, J84111, J84112, J84113, J84114, J84115, J842, J84116, J84117, J8481, J8482, J84841, J84842, J8483, J84843, J84848, J849
Idiopathic pulmonary fibrosis	J84112
Malignant neoplasm of bronchus and lung	C34
Pneumonia	J12-J18

*Comorbidities and/or symptoms were defined by the presence of a single *ICD-10-CM* code in any position from
inpatient, outpatient, or office visits claims.
ICD-10-CM, International Classification of Diseases, 10th Revision, Clinical Modification.

Table S2 Subgroup analysis among patients with hospitalisations in the COPD group (univariate analysis)

	**COPD with NTM-PD**	**COPD without NTM-PD**	***P* value***
All-cause hospitalisation, n	98	186	
Length of stay (days)			0.42
Mean (SD)	11.9 (12.8)	13.4 (15.8)	
Median (Q1, Q3)	8 (3.6, 14.9)	9 (4.5, 15.9)	
Number of hospitalisations per patient			0.6
Mean (SD)	1.62 (1.24)	1.62 (1.12)	
Median (Q1, Q3)	1 (1–2)	1 (1–2)	
Respiratory-related hospitalisation, n	56	71	
Length of stay (days)			0.45
Mean (SD)	14.5 (12.0)	17.1 (17.2)	
Median (Q1, Q3)	9.8 (6.6, 17.7)	13 (7, 20.8)	
Number of hospitalisations per patient			0.97
Mean (SD)	1.68 (1.19)	1.66 (1.16)	
Median (Q1, Q3)	1 (1–2)	1 (1–2)	
COPD-related hospitalisation, n	30	35	
Length of stay (days)			0.17
Mean (SD)	12.9 (11.3)	19.7 (21.2)	
Median (Q1, Q3)	10.5 (6.3, 15)	13 (8, 24)	
Number of hospitalisations per patient			0.61
Mean (SD)	1.57 (1.14)	1.80 (1.43)	
Median (Q1, Q3)	1 (1–2)	1 (1–2)	

**P* values were based on Wilcoxon rank-sum test.
COPD, chronic obstructive pulmonary disease; NTM-PD, nontuberculous mycobacterial pulmonary disease; Q, quartile; SD, standard deviation.
